# Prevalence and Associated Factors of Excessive Recreational Screen Time Among Colombian Children and Adolescents

**DOI:** 10.3389/ijph.2022.1604217

**Published:** 2022-02-23

**Authors:** Silvia A. González, Olga L. Sarmiento, Alberto Florez-Pregonero, Peter T. Katzmarzyk, Jean-Philippe Chaput, Mark S. Tremblay

**Affiliations:** ^1^ School of Epidemiology and Public Health, Faculty of Medicine, Ottawa, ON, Canada; ^2^ Healthy Active Living and Obesity Research Group, Children's Hospital of Eastern Ontario Research Institute, Ottawa, ON, Canada; ^3^ School of Medicine, Universidad de los Andes, Bogotá, Colombia; ^4^ School of Education, Pontificia Universidad Javeriana, Bogotá, Colombia; ^5^ Pennington Biomedical Research Center, Baton Rouge, LA, United States; ^6^ School of Epidemiology and Public Health, Faculty of Medicine, University of Ottawa, Ottawa, ON, Canada

**Keywords:** children, surveillance, adolescents, screen exposure, sedentary behaviors

## Abstract

**Objectives:** Excessive recreational screen time (RST) is associated with detrimental effects for physical, psychological and cognitive development. This article aims to describe the prevalence of excessive RST among Colombian preschoolers, children and adolescents and explore its factors associated.

**Methods:** We analyzed data from the National Survey of Nutrition 2015. The sample included 4,503 preschoolers, 5,333 school-aged children and 6,623 adolescents. Poisson regression models with robust variance were conducted to estimate prevalence ratios and determine associated factors of excessive RST.

**Results:** Fifty percent of preschoolers, 61% of school-aged children and 73% of adolescents in Colombia had excessive RST. Positive associations were observed with the availability of TV in the child’s bedroom, the availability of video games at home, and eating while using screens. A negative association with rural area was observed for all age groups.

**Conclusion:** The majority of Colombian children and adolescents have excessive RST. Younger preschoolers, older school-aged children, wealthiest children and those from urban areas should be targeted by interventions to decrease RST. These interventions should promote limiting the availability of electronic devices in children’s bedrooms and not eating in front of screens.

## Introduction

Sedentary behavior, defined by the Sedentary Behavior Research Network as “any waking behavior characterized by an energy expenditure ≤1.5 metabolic equivalents (METs), while in a sitting, reclining or lying posture” [1] has been identified as an important risk factor for poor health. Excessive time spent in sedentary behaviors, mainly recreational screen time (RST), has been associated with detrimental effects on health and development in children including unfavorable body composition, lower fitness, higher cardiometabolic risk scores, greater energy intake and poorer diet quality, lower measures of self-esteem and prosocial behaviors, lower quality of life and lower academic achievement [2–5]. Concerningly, there is a large proportion of children and adolescents around the world who spend excessive time in sedentary pursuits, and may have a higher risk of those conditions [6, 7].

The high prevalence of excessive sedentary time globally is likely a result of greater access to labor-saving technologies, motorized vehicles and screen-based entertainment which have led to a shift from active lifestyles to greater involvement in activities that require lower energy expenditure [4, 8]. In response to this situation, countries like Canada and Australia, and recently the World Health Organization, have developed public health guidelines to promote a reduction in sedentary behaviors [5, 9, 10]. These guidelines focus their message on the reduction of RST such as watching TV, playing videogames or using computers or tablets for non-school related purposes, as these are some of the main sedentary behaviors with important implications for health [4]. Using the cut-off point of Australian and Canadian sedentary behavior guidelines for children and adolescents, global data indicate that the prevalence of meeting sedentary behavior guidelines ranges from 7% in Estonia and China to 85% in Bangladesh [6, 11–13].

An important element to advance in the design of interventions to reduce sedentary behaviors is to identify the main factors positively or negatively associated to these behaviors. Sedentary behaviors can be driven by factors from multiple environments or at different levels of influence, therefore, these factors can be better understood and addressed from a context-specific approach, such as a socio-ecological framework of health behaviors [14]. The socio-ecological model applied to sedentary behaviors states that there are characteristics or variables at the individual, social, organizational/community, environmental, and policy level that can influence these behaviors [14]. Using a socio-ecological approach, a systematic review identified certain consistent positively associated factors at the individual, interpersonal and environmental levels, such as age, weight, Afro-American ethnicity, food consumption in front of the TV and built environment variables such as playground density and greater access to play and sports infrastructure [15]. However, the associations with sex and socioeconomic status found in this review were inconsistent, and evidence from low- and middle-income countries was absent.

In Colombia, screen time has been included as a relevant indicator for nutrition surveillance since 2005 [16], and its assessment has evolved over time. In the first version of the National Survey of Nutrition (ENSIN for its name in Spanish), screen time was assessed among 5–12 year-old children as TV viewing and videogame use [17]. In the 2010 survey, the same indicator was included and extended to adolescents aged 13–17 years [18]. For the most recent 2015 survey the indicator was modified to include time spent using other devices such as tablets, portable videogame devices and mobile phones for recreational purposes, and extended the population of interest to include preschool children in addition to the previously included age groups. Therefore, this is the first study in Colombia and among the first in Latin America that examines engagement in excessive RST in a nationally representative sample of pre-schoolers, children and adolescents between 3 and 17 years old. To the best of our knowledge, this is the first time that 2015 ENSIN data on RST among children and adolescents is presented. This initial approach to a broader range of recreational screen-based activities in a national sample with a larger age range provides a unique opportunity to help inform public health policies that contribute to decrease sedentary behavior in Colombia. In this context, this paper aims to describe the prevalence of excessive RST among Colombian preschoolers, children and adolescents and explore the factors associated with excessive RST within a socio-ecological framework.

## Methods

### Study Design and Participants

We analyzed data from the sedentary behavior component of ENSIN 2015. ENSIN is a cross-sectional population health survey with national representativeness of urban and rural areas of Colombia [19]. The sample of this survey was selected with a stratified, multistage probability cluster sampling design. The sub-sample for the sedentary behavior component included 4,503 preschoolers (3–5 years), 5,333 school-aged children (6–12 years) and 6,623 adolescents (13–17 years). The survey was administered by the Colombian Institute of Family Welfare.

### Data Collection

Data were collected by trained nutritionists in the household setting between December 2015 and November 2016, using computer-assisted personal interview technology with a data collection system specifically designed for ENSIN. Data collection teams were regularly accompanied by researchers for quality control purposes. For preschoolers and school-aged children, one of the parents or the main caregiver responded to the survey, while adolescents self-reported their responses. Parents provided signed informed consent and children between 12 and 17 years provided informed assent before conducting the survey. The ENSIN protocol was approved by the *Profamilia* Institutional Review Board on Research involving Human Subjects and the Colombian National Institutes of Health (file number 2-2015, 26 February 2015). The secondary data analyses were approved by the University of Ottawa (file number H-06-19-3564). Data collection details according to the type of variables (dependent vs. potential associated factors) are described below.

#### Dependent Variables

Among preschoolers, RST was assessed with the Questionnaire for the Measurement of Physical Activity and Sedentary Behaviors in preschool to fourth grade children (C-MAFYCS), which has low internal consistency for the sedentary behavior scale (Cronbach’s alpha = .31) and moderate to good reproducibility mainly for items such as computer and videogame use (Kappa = .71 and .62, respectively) [20]. Engagement in screen-based activities was assessed with three questions: “During the last 7 days, did (name of the child) watch television, movies or videos in the TV? During the last 7 days, did (name of the child) play or listen to music in the computer? And during the last 7 days, did (name of the child) play with consoles such as X-Box, Play-Station, manual videogames such as Nintendo DS, etc., play with a cellphone, smartphone, iPad or other tablets?” Each of these questions was informed with the amount of time spent in each activity with the following questions: How much time did he/she spend on weekdays? And how much time did he/she spend on weekends? (for each day) The total time in all screen-based activities per weekdays and weekend days was estimated in minutes and the average screen time per day, per weekday and per weekend day was calculated. A dichotomous variable to indicate if the preschooler achieved excessive RST was calculated (excessive vs. not excessive RST). Excessive RST was defined as engaging in more than 1 h of RST per day among preschoolers under 5 years of age or more than 2 h of RST per day among 5-year-olds, in accordance with the WHO and the Canadian sedentary behavior guidelines [9, 21].

Among school-aged children and adolescents, RST was assessed with the following questions adapted from the US Youth Risk Behavior Surveillance System (YRBSS) [22]: “On an average school day or weekday, how many hours did (name of the child)/you watch TV?; On an average school day or weekday, how many hours did (name of the child)/you play video games or use a computer for something that is not schoolwork?; On a weekend day, how many hours did (name of the child)/you watch TV? And, on a weekend day, how many hours did (…) play video games or use a computer for something that is not schoolwork?” The question inquiring about videogames and computer use included the following examples of devices and activities to consider: Xbox, PlayStation, iPod, iPad or other tablet, smartphone, cellphone, YouTube, Facebook or other social media and internet. The response options were: 1) Did not watch TV/played videogames or used the computer, 2) less than 1 h, 3) between 1 and 1:59 h, 4) between 2 and 2:59 h, 5) between 3 and 3:59 h, 6) between 4 and 4:59 h, and 7) 5 h or more. A screen time score was calculated converting these categories as follows: 1 and 2 = 0 h, 3 = 1 h, 4 = 2 h, 5 = 3 h, 6 = 4 h and 7 = 5 h. With these values we calculated a weighted mean score of daily screen time, as previously estimated in other studies [23]. A dichotomous variable to indicate excessive RST was created using a cut-off of more than 2 h of screen time, according to the sedentary behavior guidelines in the Canadian 24-h movement guidelines [9].

#### Potential Associated Factors

We selected variables that were potentially relevant for the analysis of RST at different levels according to the socio-ecological framework of sedentary behaviors [14].

##### Intrapersonal Level

Sex (female vs. male), age, ethnicity (Afro-Colombian vs. Indigenous vs. Other ethnic identity), overweight (yes vs. no), physical activity program participation (yes vs. no), physical activity (meeting vs. not meeting the WHO physical activity guidelines), and food frequency consumption variables (yes vs. no) were included at the individual level. Sociodemographic variables (i.e., sex, age, ethnicity) were measured with the Household Questionnaire designed for ENSIN [19]. Overweight (including obesity) was ascertained based on body mass index, calculated from objective measurements of height and weight, and categorized according to WHO growth standards and reference tables [24, 25]. Physical activity program participation (organized activities, sports clubs, and Ciclovías for school-aged children and adolescents, and programs at the school and community levels among adolescents) was assessed with questions designed for the Physical Activity and Sedentary Behavior Questionnaire for ENSIN [19]. Physical activity was proxy-reported (by parents or caregivers) for pre-schoolers and school-aged children and self-reported by adolescents. For pre-schoolers, physical activity was assessed with the C-MAFYCS questionnaire, and for school-aged children and adolescents it was assessed with a question adapted from the YRBSS. A dichotomous variable to indicate if the child/adolescent met the physical activity guidelines was created, using a cut-off of 180 min of physical activity per day with at least 60 min of energetic play for preschool children, and 7 days of being active at least 60 min per day for school-age children [26]. More details on the physical activity assessment are described elsewhere [27]. Food consumption variables were measured with the Food Frequency Questionnaire designed for ENSIN [19] and specific food groups were incorporated in the analysis including: charcuterie products, sodas, snacks and fried foods at least three times per week; fast food consumption at least once per week; and daily consumption of candy. We also assessed usual food consumption while using screens during the last month.

##### Household Level

At the household level, we included wealth index, area of residence (urban vs. rural), and TV and video game device availability in the child’s bedroom. To estimate wealth index, data on asset ownership, availability of public utilities and materials used for housing construction were collected in the socio-demographic section of the survey. Then, the index was calculated using a principal component approach and categorized into quartiles. TV and videogame availability were assessed with questions adapted from the Neighborhood Impact on Kids (NIK) survey [28].

##### Environmental and Community Level

At the community level we included parks availability in the neighbourhood and perceived safety in the parks, which were assessed with questions adapted from the NIK survey [28]. For the natural environment level, we included the geographic region where the children lived at the time of the survey (Orinoquia-Amazonia vs. Atlantic vs. Central vs. Eastern vs. Pacific vs. Capital District).

### Statistical Analysis

Descriptive statistics (means, standard deviations, and frequencies) were estimated for demographic and contextual characteristics of the sample and chi-squared tests were conducted to determine differences between groups. Poisson regression models with robust variance were conducted to estimate prevalence ratios (PR) and determine associated factors of excessive RST. Three types of models were run for each age group: 1) bivariate models with potential associated factors, 2) multivariable model 1 included the variables available for the whole sample that showed at least marginally significant associations (*p* < .10) and were adjusted for age and sex and 3) multivariable model 2 included the food consumption and program participation variables that had *p*-values <.10 in the bivariate analyses and were conducted as a sub-analysis with a smaller sample size. The reason for running two different multivariable models was that not all the participants had the same covariates available, as some components of ENSIN had different selection probabilities. Therefore, multivariable model 1 included the whole sample, and multivariable model 2 explored additional variables with the sub-sample of participants that also had food consumption and programs attendance data available. Variables with *p*-values <.05 in the multivariable models were considered associated factors of excessive RST. We used STATA 14.0 (StataCorp, College Station, TX, United States) for the analyses, with the survey (SVY) module for complex samples to take into account the clustering of data and the characteristics of the study design, sample weights and missing data. Respondents with missing data on the main variables of interest were excluded from the study (4 observations). Missing data on covariates are described in the footnotes for Tables 1 and 2.

**TABLE 1:** Sociodemographic and lifestyle characteristics of 4,503 preschoolers from Colombia. National Survey of Nutrition, Colombia, 2015.

	Preschool children									
Sociodemographic and other potentially relevant variables for screen-time	Total sample			Males			Females			
	*n* ^a^	%	SE	*N*	%	SE	*n*	%	SE	*p*-value
Sex										
Female	2,195	48.5	1.21		NA			NA		
Male	2,308	51.5	1.21		NA			NA		
Age (years)										
3–4	2,998	70.1	1.03	1,515	70.4	1.47	1,483	69.8	1.35	.746
5	1,505	29.9	1.03	793	29.6	1.47	712	30.2	1.35	
Ethnicity										
Afro-colombian	441	9.1	.78	222	8.2	.81	219	10.0	1.11	.178
Indigenous	480	4.7	.63	246	4.5	.71	234	4.9	.71	
Other	3,544	86.2	.84	1819	87.3	.94	1725	85.1	1.16	
Overweight										
Yes	505	11.0	.76	300	13.4	1.22	205	8.5	.82	.001
No	3,856	89.0	.76	1,934	86.6	1.22	1,922	91.5	.82	
Physically active^b^										
Yes	1,075	24.4	1.1	635	28.6	1.6	440	19.92	1.39	<.001
No	3,428	75.6	1.1	1,673	71.4	1.6	1755	80.08	1.39	
Food intake^c^										
Charcuterie 3 times per week or more										
Yes	1,077	34.8	1.49	557	35.4	1.83	520	34.2	2.10	.625
No	1,950	65.2	1.49	1,012	64.6	1.83	938	65.8	2.10	
Sodas 3 times per week or more										
Yes	1,393	45.6	1.38	731	46.4	1.98	662	44.8	1.83	.560
No	1,780	54.4	1.38	888	53.6	1.98	892	55.2	1.83	
Snacks 3 times per week or more										
Yes	1,332	44.3	1.40	689	44.4	1.90	643	44.1	1.78	.914
No	1,908	55.8	1.40	956	55.6	1.90	952	55.9	1.78	
Fried foods 3 times per week or more										
Yes	2,008	53.4	1.24	1,017	52.2	1.77	991	54.6	1.68	.337
No	1,751	46.6	1.24	908	47.8	1.77	843	45.5	1.68	
Fast food once per week or more										
Yes	213	9.9	.93	112	10.4	1.30	101	9.5	1.31	.652
No	1,599	90.1	.93	804	89.6	1.30	795	90.5	1.31	
Candy once per day or more										
Yes	1,426	42.6	1.21	711	40.2	1.67	715	45.1	1.71	.044
No	2,168	57.4	1.21	1,136	59.8	1.67	1,032	54.9	1.71	
Usually eats while using screens										
Yes	2,463	62.5	1.27	1,274	62.3	1.62	1,189	62.7	1.84	.860
No	1,683	37.5	1.27	859	37.7	1.62	824	37.3	1.84	
Wealth Quartiles										
First	2,371	41.1	1.31	1,197	40.4	1.58	1,174	41.9	1.90	.655
Second	1,086	26.3	1.18	575	27.6	1.50	511	24.9	1.56	
Third	713	20.3	1.22	365	19.6	1.34	348	21.0	2.04	
Fourth	333	12.3	1.01	171	12.4	1.35	162	12.2	1.35	
Area										
Urban	3,208	72.4	1.20	1,631	71.8	1.47	1,577	72.9	1.71	.593
Rural	1,293	27.6	1.20	675	28.2	1.47	618	27.1	1.71	
TV available at the child’s bedroom										
Yes	1734	42.3	1.15	906	44.3	1.58	828	40.1	1.55	.057
No	2,767	57.7	1.15	1,400	55.7	1.58	1,367	59.9	1.55	
Video games devices availability										
Yes	554	14.0	.84	295	13.7	1.12	259	14.2	1.32	.788
No	3,947	86.0	.84	2011	86.3	1.12	1936	85.8	1.32	
Parks availability in the neighborhood										
Yes	2,376	55.4	1.37	1,273	56.9	1.70	1,103	53.8	1.94	.187
No	2,125	44.6	1.37	1,033	43.1	1.70	1,092	46.3	1.94	
Safety perception^d^										
Is safe to play in the park	1,793	75.7	1.57	977	77.3	1.97	816	74.0	2.22	.233
Is not safe to play in the park	583	24.3	1.57	296	22.7	1.97	287	26.0	2.22	
Geographic región										
Atlantic	932	24.4	1.03	490	25.0	1.26	442	23.7	1.44	
Eastern	799	17.0	1.24	401	16.2	1.42	398	17.8	1.61	
Orinoquia-Amazonia	775	3.9	.27	395	3.9	.53	380	4.0	.47	
Capital District	318	14.0	1.30	161	14.3	1.46	157	13.7	2.14	
Central	969	24.2	.93	497	24.9	1.35	472	23.5	1.34	
Pacific	710	16.5	.70	364	15.8	.94	346	17.3	1.17	

a The total sample size for preschoolers was 4,503 children. However the following variables had missing values: ethnicity = 38, BMI = 142, area, TV availability, video games availability and parks availability = 2.

b Physically active is defined as meeting the WHO physical activity guidelines using a cut-off of 180 min of physical activity per day with at least 60 min of energetic play.

c Food intake variables are available only for the sub-sample selected for the food intake component of the survey, therefore the sample sizes are smaller.

d Safety perception was assessed only among those who reported having a park in their neighborhood.

n, sample size; SE, standard error; NA, not applicable.

**Table 2:** Sociodemographic and lifestyle characteristics of 11,956 children and adolescents from Colombia. National Survey of Nutrition, Colombia, 2015.

	School aged children										Adolescents									
Sociodemographic and other potentially relevant variables for screen-time	Total sample			Males			Females				Total sample			Males			Females			
	*n* ^a^	%	SE	*n*	%	SE	*N*	%	SE	*p*-value	*n* ^b^	%	SE	*n*	%	SE	*n*	%	SE	*p*-value
Sex																				
Female	2,556	48.0	1.12	NA			NA			NA	3,329	49.6	.99	NA			NA			NA
Male	2,777	52.0	1.12	NA			NA				3,294	50.4	.99	NA			NA			
Age (years)																				
6–9	3,015	58.8	1.10	1,535	58.1	1.47	1,480	59.5	1.73	.56	NA			NA			NA			NA
10–12	2,318	41.2	1.10	1,242	41.9	1.47	1,076	40.5	1.73		NA			NA			NA			
13–15	NA			NA			NA			NA	4,007	60.3	.99	2,009	59.0	1.45	1,998	61.7	1.36	.179
13–17	NA			NA			NA				2,616	39.7	.99	1,285	41.0	1.45	1,331	38.3	1.36	
Ethnicity																				
Afro-colombian	452	9.8	.93	233	9.6	1.09	219	10.1	1.06	.834	557	9.2	.94	316	9.7	1.19	241	8.7	.96	.238
Indigenous	671	5.9	.78	333	5.7	1.23	338	6.1	1.06		652	4.5	.80	261	4.0	.83	391	5.0	.89	
Other	4,165	84.3	1.11	2,191	84.8	1.48	1974	83.8	1.39		5,361	86.3	.82	2,689	86.3	1.05	2,672	86.3	.99	
Overweight																				
Yes	1,255	25.8	1.15	668	27.1	1.60	587	24.5	1.51	.221	1,234	18.1	.75	447	14.9	.98	787	21.4	1.11	<.001
No	3,894	74.2	1.15	2019	73.0	1.60	1875	75.5	1.51		5,120	81.9	.75	2,692	85.2	.98	2,428	78.6	1.11	
Participation in organized activities and programs^c^																				
Ciclovias^d^																				
Yes	83	2.0	.45	51	1.9	.410	32	2.0	.84	.925	459	15.4	1.15	274	20.7	1.86	185	10.1	1.13	<.001
No	5,250	98.0	.45	2,726	98.1	.410	2,524	98.0	.84		2,999	84.6	1.15	1,408	79.3	1.86	1,591	89.9	1.13	
Community physical activity programs (e.g. aerobics)																				
Yes	NA			NA			NA			NA	797	20.2	1.19	289	15.9	1.25	508	23.9	1.77	<.001
No	NA			NA			NA				2,840	79.8	1.19	1,351	84.1	1.25	1,489	76.1	1.77	
Physical activity programs at school																				
Yes	NA			NA			NA			NA	1889	47.9	1.29	984	50.5	1.68	905	45.4	1.87	<.001
No	NA			NA			NA				2006	52.1	1.29	912	49.5	1.68	1,094	54.6	1.87	
Sport Clubs																				
Yes	2,237	41.7	1.18	1,477	54.3	1.75	760	28.1	1.50	<.001	1,104	27.9	1.16	759	38.0	1.72	345	16.7	1.28	<.001
No	3,096	58.3	1.18	1,300	45.7	1.75	1796	72.0	1.50		2,671	72.1	1.16	1,228	62.0	1.72	1,443	83.3	1.28	
Organized groups (dance, martial arts, etc)																				
Yes	484	8.3	.69	171	5.4	.73	313	11.4	1.13	<.001	719	19.9	1.18	249	15.9	1.52	470	23.1	1.70	.002
No	4,849	91.7	.69	2,606	94.6	.73	2,243	88.6	1.13		2,778	80.1	1.18	1,300	84.1	1.52	1,478	76.9	1.70	
Physically active^e^																				
Yes	1722	31.1	1.40	1,042	35.8	1.91	680	26.0	1.62	<.001	848	13.3	.76	605	18.8	1.12	243	7.6	.87	<.001
No	3,611	68.9	1.40	1735	64.2	1.91	1876	74.0	1.62		5,775	86.8	.76	2,689	81.2	1.12	3,086	92.4	.87	
Food intake^f^																				
Yes	1,256	37.2	1.33	692	40.0	1.93	564	34.1	1.92	.039	1,878	39.3	1.08	966	40.7	1.63	912	37.8	1.52	.212
No	2,422	62.9	1.33	1,249	60.0	1.93	1,173	65.9	1.92		3,018	60.7	1.08	1,527	59.3	1.63	1,491	62.2	1.52	
Sodas 3 times per week or more																				
Yes	1,965	51.5	1.64	997	51.8	2.07	968	51.1	2.15	.791	3,420	61.9	1.04	1,832	65.5	1.29	1,588	58.0	1.48	<.001
No	2,045	48.5	1.64	1,108	48.2	2.07	937	48.9	2.15		2,159	38.1	1.04	1,022	34.5	1.29	1,137	42.0	1.47	
Snacks 3 times per week or more																				
Yes	1,753	50.0	1.52	916	52.3	2.07	837	47.6	1.81	.059	2,527	49.5	1.18	1,272	49.1	1.60	1,255	49.9	1.54	.706
No	2,194	50.0	1.52	1,133	47.7	2.07	1,061	52.4	1.81		2,763	50.5	1.18	1,365	50.9	1.60	1,398	50.1	1.54	
Fried foods three times per week or more																				
Yes	2,584	56.2	1.37	1,378	56.9	1.96	1,206	55.6	1.73	.612	3,766	60.0	1.09	1904	60.9	1.38	1862	59.0	1.49	.333
No	2029	43.8	1.37	1,032	43.2	1.96	997	44.4	1.73		2,370	40.0	1.09	1,172	39.1	1.38	1,198	41.0	1.49	
Fast food once per week or more																				
Yes	329	12.4	1.21	165	11.7	1.60	164	13.2	1.87	.556	1,041	25.1	1.14	553	26.1	1.60	488	24.1	1.46	.337
No	2,179	87.6	1.21	1,126	88.3	1.60	1,053	86.9	1.87		2,986	74.9	1.14	1,450	73.9	1.60	1,536	75.9	1.46	
Candy once per day or more																				
Yes	2,136	53.9	1.50	1,064	52.7	2.01	1,072	55.1	1.83	.338	2,692	51.3	1.24	1,284	49.4	1.71	1,408	53.2	1.60	.091
No	2,277	46.2	1.50	1,233	47.3	2.01	1,044	45.0	1.83		2,786	48.7	1.24	1,420	50.6	1.71	1,366	46.8	1.60	
Usually eats while using screens																				
Yes	3,208	68.3	1.24	1,651	66.2	1.83	1,557	70.6	1.62	.074	4,711	74.7	1.02	2,353	74.2	1.22	2,358	75.2	1.29	.497
No	1808	31.7	1.24	969	33.8	1.83	839	29.4	1.62		1829	25.3	1.02	906	25.8	1.22	923	24.8	1.29	
Wealth Quartiles																				
First	2,867	39.2	1.56	1,479	39.0	2.06	1,388	39.4	1.78	.575	3,342	38.1	1.31	1,658	38.9	1.49	1,684	37.3	1.71	.673
Second	1,289	25.6	1.20	708	25.8	1.49	581	25.4	1.64		1,537	23.3	.96	770	23.6	1.21	767	23.0	1.21	
Third	763	19.7	1.03	382	18.7	1.35	381	20.7	1.44		1,097	21.2	.94	545	20.9	1.27	552	21.5	1.24	
Fourth	414	15.5	1.14	208	16.4	1.59	206	14.5	1.34		647	17.5	1.11	321	16.7	1.39	326	18.2	1.41	
Area																				
Urban	4,016	73.7	1.35	2,087	74.0	1.77	1929	73.4	1.57	.782	4,816	73.8	1.29	2,300	73.2	1.56	2,516	74.4	1.44	.436
Rural	1,317	26.3	1.35	690	26.0	1.77	627	25.6	1.57		1,807	26.2	1.29	994	26.8	1.57	813	25.6	1.44	
TV available at the child’s bedroom																				
Yes	1807	39.6	1.32	921	39.0	1.79	886	40.3	1.76	.601	2,363	41.6	1.07	1,193	42.0	1.50	1,170	41.3	1.41	.723
No	3,525	60.4	1.32	1855	61.0	1.79	1,670	59.7	1.76		4,259	58.4	1.07	2,100	58.0	1.50	2,159	58.7	1.41	
Video games devices availability																				
Yes	1,452	33.7	1.28	782	35.2	1.85	670	32.0	1.70	.208	3,835	62.2	1.01	1904	61.7	1.39	1931	62.7	1.30	.552
No	3,881	66.3	1.28	1995	64.8	1.85	1886	68.0	1.70		2,788	37.8	1.01	1,465	38.3	1.39	1,398	37.3	1.30	
Parks availability in the neighborhood																				
Yes	3,121	61.4	1.54	1,650	62.6	1.89	1,471	60.1	1.90	.263	4,203	66.8	1.12	2,179	69.1	1.32	2024	64.5	1.43	.006
No	2,212	38.6	1.54	1,127	37.4	1.89	1,085	39.9	1.90		2,420	33.2	1.12	1,115	30.9	1.32	1,305	35.5	1.43	
Safety perception^g^																				
Is safe to play in the park	2,371	72.1	1.58	1,271	72.7	2.12	1,100	71.4	2.14	.652	3,130	72.2	1.22	1718	76.9	1.64	1,412	67.2	1.79	<.001
Is not safe to play in the park	750	27.9	1.58	379	27.3	2.12	371	28.6	2.14		1,073	27.8	1.22	461	23.2	1.64	612	32.8	1.79	
Geographic region																				
Atlantic	786	23.5	1.54	418	23.4	2.05	368	23.6	1.67	.759	1,340	24.4	1.06	778	25.6	1.43	562	23.2	1.23	.682
Eastern	809	17.9	1.88	409	17.1	2.05	400	18.8	1.91		1,149	18.0	1.57	587	17.7	1.63	562	18.4	1.77	
Orinoquia-Amazonia	1,560	3.3	.24	802	3.2	.32	758	3.4	.25		1,248	3.1	.20	440	3.2	.32	808	3.0	.25	
Capital District	367	14.5	1.39	193	14.2	1.85	174	14.7	1.60		382	12.3	1.12	202	11.9	1.64	180	12.7	1.27	
Central	1,193	24.4	1.22	630	25.4	1.68	563	23.2	1.31		1,636	24.4	.90	807	23.7	1.26	829	25.1	1.19	
Pacific	618	16.5	.93	325	16.7	1.19	293	16.2	1.22		868	17.8	.83	480	17.8	1.04	388	17.7	1.13	

a The total sample size for school-aged children was 5,333 children. However the following variables had missing values: ethnicity = 45, BMI = 184 and TV availability = 1.

b The total sample size for adolescents was 6,623. However the following variables had missing values: ethnicity = 53, BMI = 269, and TV availability = 1.

c Participation in organized activities and programs was assessed only among school-age children and adolescents who reported knowing those programs.

d Ciclovias are defined as a program that closes the streets to motorized vehicles, usually Sundays and holidays, for recreational and exercise purposes.

e Physically active is defined as meeting the WHO physical activity guidelines.

f Food intake variables are available only for the sub-sample selected for the food intake component of the survey, therefore the sample sizes are smaller.

g Safety perception was assessed only among those who reported having a park in their neighborhood.

*n*, sample size; SE, standard error; NA, not applicable.

## Results

Sociodemographic characteristics of the sample and other descriptive statistics for this analysis are presented in Tables 1, 2. Approximately 48% of the sample for each age group were females. About 9% and 5% of the children and adolescents were Afro-Colombian or indigenous, respectively. Overweight prevalence was 11.0% for preschoolers, 25.8% among school-aged children, and 18.1% among adolescents. Male preschoolers had a higher prevalence of overweight compared to females, while female adolescents had a higher prevalence of overweight compared to male adolescents. Low proportions of active children were observed across all age groups and were lower among females. More than 60% of preschoolers and school-aged children, and over 70% of adolescents reported eating in front of screens. Over 70% of children and adolescents lived in urban areas, about 40% had a TV available in their bedroom, and availability of video game devices ranged from 14% among preschoolers to more than 60% among adolescents (Tables 1, 2).

### Prevalence of Excessive Recreational Screen-Time

Table 3 presents the overall prevalence of excessive RST and the prevalence according to potentially relevant variables. We found that 50% of preschoolers, 61% of school-aged children and 73% of adolescents in Colombia had excessive RST (Table 3). This prevalence did not differ by sex. Among preschoolers, younger children (3–4 years) had a greater prevalence of excessive RST, while in school-aged children this prevalence was higher for older children (10–12 years). Indigenous preschoolers, children and adolescents had a lower prevalence of excessive RST. Among school-aged children, those with overweight or who were physically inactive, had greater prevalences of excessive RST.

**TABLE 3:** Prevalence of excessive recreational screen-time among Colombian preschoolers, school-aged children and adolescents. National Survey of Nutrition, Colombia, 2015.

Sociodemographic and other potentially relevant variables for screen-time	Preschool children^a^				School aged children^b^				Adolescents^b^			
	P (%)	CI	SE	*p*-value	P (%)	CI	SE	*p*-value	P (%)	CI	SE	*p*-value
Total	50.1	(47.5–52.7)	1.294		61.0	(58.4–63.6)	1.303		72.5	(70.5–74.4)	.964	
Sex												
Female	50.5	(46.8–54.2)	1.856	.74	60.2	(56.7–63.6)	1.724	.52	73.0	(70.2–75.5)	1.332	.636
Male	49.7	(46.4–53.1)	1.661		61.7	(58.1–65.2)	1.778		72.1	(69.2–74.7)	1.383	
Age												
3–4	56.4	(53.4–59.3)	1.494	<.001	NA			NA	NA			NA
5	35.5	(31.6–39.5)	1.978		NA				NA			
6–9	NA			NA	57.2	(54.0–60.4)	1.597	<.001	NA			NA
10–12	NA				66.4	(62.8–69.7)	1.723		NA			
13–15	NA			NA	NA			NA	72.5	(70.1–74.8)	1.176	.972
13–17	NA				NA				72.5	(69.5–75.4)	1.458	
Ethnicity												
Afro-colombian	42.3	(35.4–49.5)	3.551	.001	56.0	(49.6–62.1)	3.140	<.001	73.5	(68.3–78.1)	2.449	<.001
Indigenous	37.0	(29.2–45.6)	4.147		26.6	(18.3–36.9)	4.677		54.1	(47.0–61.1)	3.543	
No ethnic identity reported	51.6	(48.7–54.5)	1.443		63.9	(61.1–66.7)	1.396		73.3	(71.1–75.4)	1.079	
Overweight												
Yes	47.0	(39.6–54.6)	3.771	.382	69.5	(65.0–73.6)	2.163	<.001	75.8	(71.7–79.4)	1.935	.153
No	50.6	(47.7–53.5)	1.443		58.1	(55.1–61.1)	1.505		72.4	(70.0–74.6)	1.143	
Physically active^c^												
Yes	51.1	(45.8–56.4)	2.653	.646	54.1	(49.7–58.4)	2.188	<.001	69.1	(63.8–74.0)	2.553	.129
No	49.8	(47.0–52.6)	1.387		64.1	(61.1–67.0)	1.456		73.0	(71.0–75.0)	.992	
Wealth Quartiles												
First (poorest)	36.7	(33.6–39.9)	1.564	<.001	45.5	(41.8–49.2)	1.859	<.001	61.5	(58.4–64.6)	1.542	<.001
Second	57.2	(52.5–61.8)	2.333		65.8	(61.2–70.2)	2.245		74.0	(69.9–77.7)	1.949	
Third	62.0	(55.5–68.2)	3.206		74.2	(69.5–78.3)	2.219		84.4	(81.1–87.1)	1.498	
Fourth (wealthiest)	60.3	(51.7–68.4)	4.208		74.5	(68.3–81.6)	3.334		80.0	(73.9–85.0)	2.771	
Area												
Urban	56.0	(52.9–59.1)	1.553	<.001	67.0	(64.1–69.8)	1.423	<.001	77.2	(75.0–79.3)	1.077	<.001
Rural	34.8	(30.6–39.1)	2.132		44.1	(40.0–48.2)	2.068		59.2	(55.2–63.1)	1.990	
TV available at the child’s bedroom												
Yes	58.5	(54.8–62.0)	1.802	<.001	71.5	(67.9–74.8)	1.709	<.001	80.8	(78.0–83.2)	1.307	<.001
No	44.1	(40.9–47.2)	1.578		54.1	(50.7–57.5)	1.697		66.6	(63.9–69.2)	1.334	
Video games devices availability												
Yes	67.1	(61.0–72.7)	2.925	<.001	79.0	(75.2–82.3)	1.779	<.001	81.6	(79.1–83.8)	1.160	<.001
No	47.4	(44.8–50.0)	1.304		51.9	(48.9–54.8)	1.493		57.6	(54.5–60.6)	1.548	
Parks availability in the neighborhood												
Yes	57.0	(53.4–60.6)	1.805	<.001	67.1	(63.8–70.3)	1.609	<.001	75.3	(72.8–77.5)	1.180	<.001
No	41.6	(38.2–45.2)	1.750		51.2	(47.6–54.9)	1.837		67.0	(64.1–69.7)	1.401	
Safety perception^d^												
Is safe to play in the park	57.0	(52.7–61.1)	2.115	.968	66.8	(63.1–70.3)	1.788	.706	75.3	(72.6–77.9)	1.311	.899
Is not safe to play in the park	57.1	(50.4–63.6)	3.336		68.0	(62.0–73.5)	2.893		75.0	(70.3–79.2)	2.231	
Geographic region												
Atlantic	39.8	(36.2–43.7)	1.881	<.001	48.0	(42.3–53.8)	2.903	<.001	65.8	(62.2–69.2)	1.736	.001
Eastern	50.1	(44.0–56.2)	3.088		67.2	(62.1–71.8)	2.432		71.3	(65.4–76.5)	2.789	
Orinoquia-Amazonia	47.6	(35.8–59.6)	6.056		52.5	(47.3–57.7)	2.609		66.8	(60.7–72.5)	2.961	
Capital District	70.6	(61.8–78.0)	4.088		72.4	(63.9–79.5)	3.926		80.4	(71.1–87.2)	4.011	
Central	54.0	(49.0–59.0)	2.508		64.5	(60.0–68.8)	2.210		78.1	(75.2–80.8)	1.396	
Pacific	42.9	(37.9–48.1)	2.571		59.3	(53.2–65.2)	3.031		70.8	(66.1–75.1)	2.261	

a Excessive recreational screen-time in pre-schoolers was defined as engaging in more than 1 hour of screen time per day among children under 5 years of age or more than 2 hours of screen time per day among 5-year-olds.

b Excessive recreational screen-time in school-aged children and adolescents was defined as engaging in more than 2 h of screen time per day.

c Physically active is defined as meeting the WHO physical activity guidelines.

d Safety perception was assessed only among those who reported having a park in their neighborhood.

P, prevalence; CI, 95% confidence intervals; SE, standard error; NA, not applicable.

The prevalence of excessive RST differed by socioeconomic status, with those in the third and fourth wealth quartiles having a higher prevalence across all age groups. Also, children and adolescents from urban areas, with TV in the bedroom, video-game devices available and those with park availability in the neighborhood had higher prevalence of excessive RST. According to geographic region, children and adolescents living in the capital district had a higher prevalence of excessive RST (Table 3).

### Associated Factors of Excessive Screen Time Among Preschoolers

**FIGURE 1:**
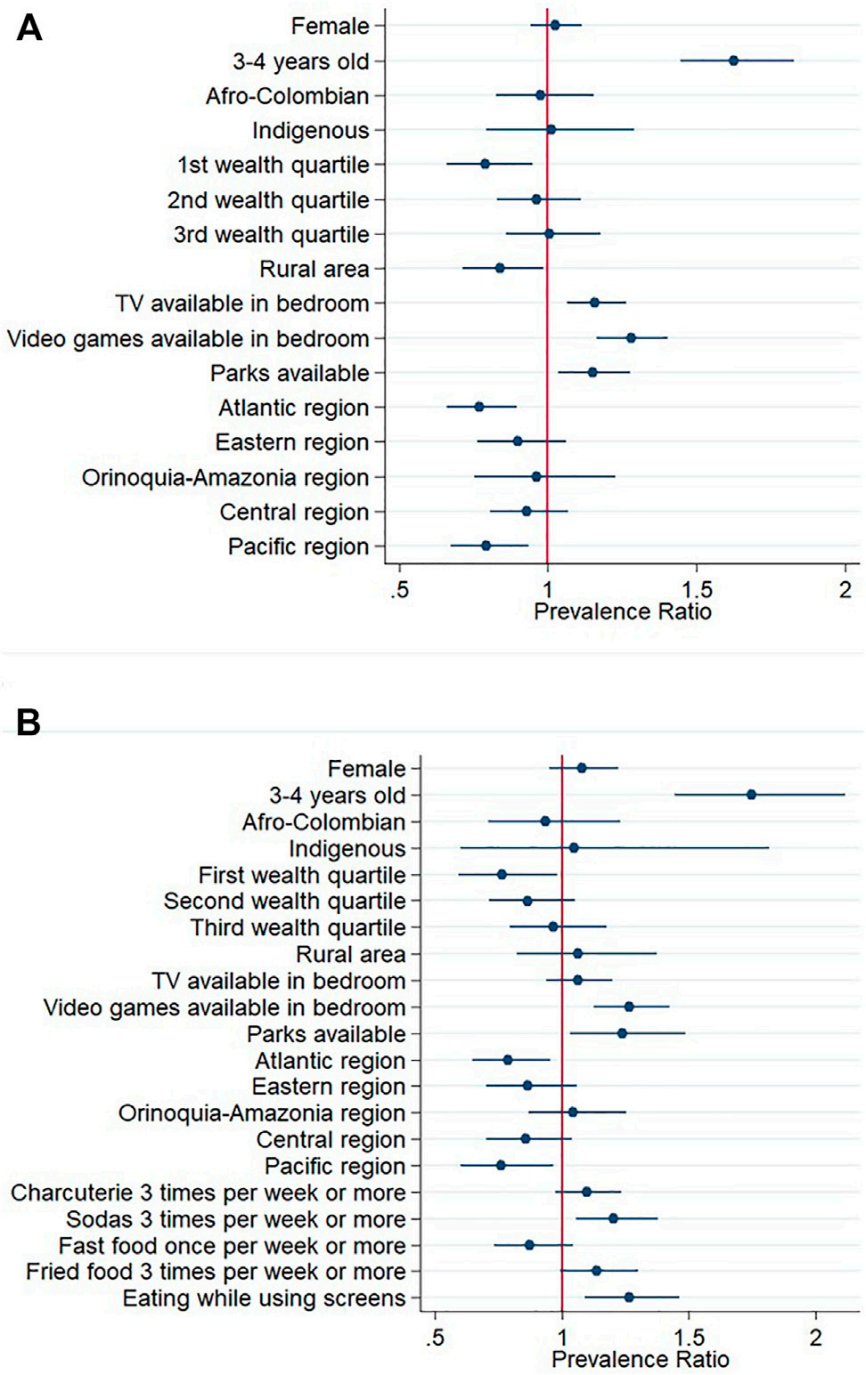
Prevalence ratios and 95% confidence intervals of associated factors of spending excessive recreational screen-time among Colombian preschoolers. Excessive screen-time was defined as spending more than 1 h of screen-time per day among children under 5 years, and engaging in more than 2 h of screen-time per day among 5-year-olds. **(A)** Mutivariable model 1 **(B)** Mutivariable model 2 including food consumption variables. Reference categories were male sex, 5 years of age, no ethnic identity reported, fourth wealth quartile, urban area, no TV availability, no video games availability, no parks availability, Capital District region, no consumption of the food items included, and no eating while using screens. National Survey of Nutrition, Colombia, 2015.

Figure 1 and Supplementary Table 1 presents the associated factors of excessive RST among preschoolers. According to multivariable model 1, younger preschoolers (3–4 year-olds), those with TV availability in their bedroom, video-game devices available, and those with parks available in their neighbourhood were more likely to have excessive RST compared to their counterparts. Also, preschoolers in the lowest wealth quartile, those living in rural areas, and those living in the Atlantic or Pacific region were less likely to have excessive RST (Figure 1A). According to multivariable model 2, soda consumption three or more times per week and eating while using screens were positively associated with having excessive RST (Figure 1B).

### Associated Factors of Excessive Screen Time Among School-Aged Children

**FIGURE 2:**
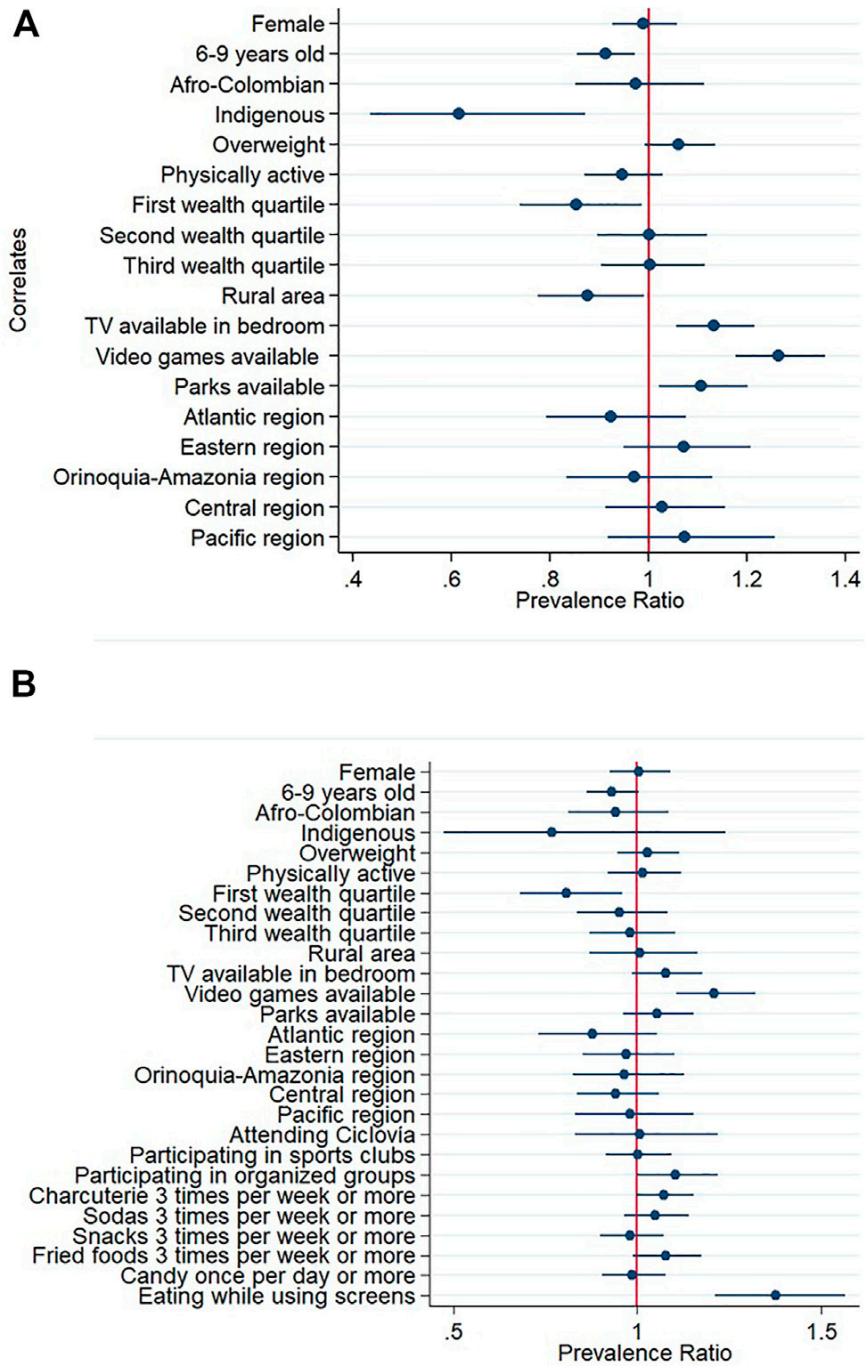
Prevalence ratios and 95% confidence intervals of associated factors of spending excessive recreational screen-time among Colombian school-aged children. Excessive screen-time was defined as spending more than 2 h of screen-time per day. **(A)** Mutivariable model 1 **(B)** Mutivariable model 2 including food consumption and program participation variables. Reference categories were male sex, 10–12 years of age, no ethnic identity reported, no overweight, not meeting physical activity guidelines, fourth wealth quartile, urban area, no TV availability, no video games availability, no parks availability, Capital District region, not attending Ciclovía, not participating in sport clubs, not participating in organized groups, no consumption of the food items included and not eating while using screens. National Survey of Nutrition, Colombia, 2015.

According to multivariable model 1, among school-aged children, those who had a TV in their bedroom or video-game device availability were more likely to have excessive RST. Younger age, Indigenous ethnicity, those in the lowest socioeconomic level, and those living in rural areas were less likely to have excessive RST (Figure 2A; Supplementary Table 2). According to multivariable model 2, there was a positive association with the usual consumption of foods in front of screens (Figure 2B; Supplementary Table 2).

### Associated Factors of Excessive Screen Time Among Adolescents

**FIGURE 3:**
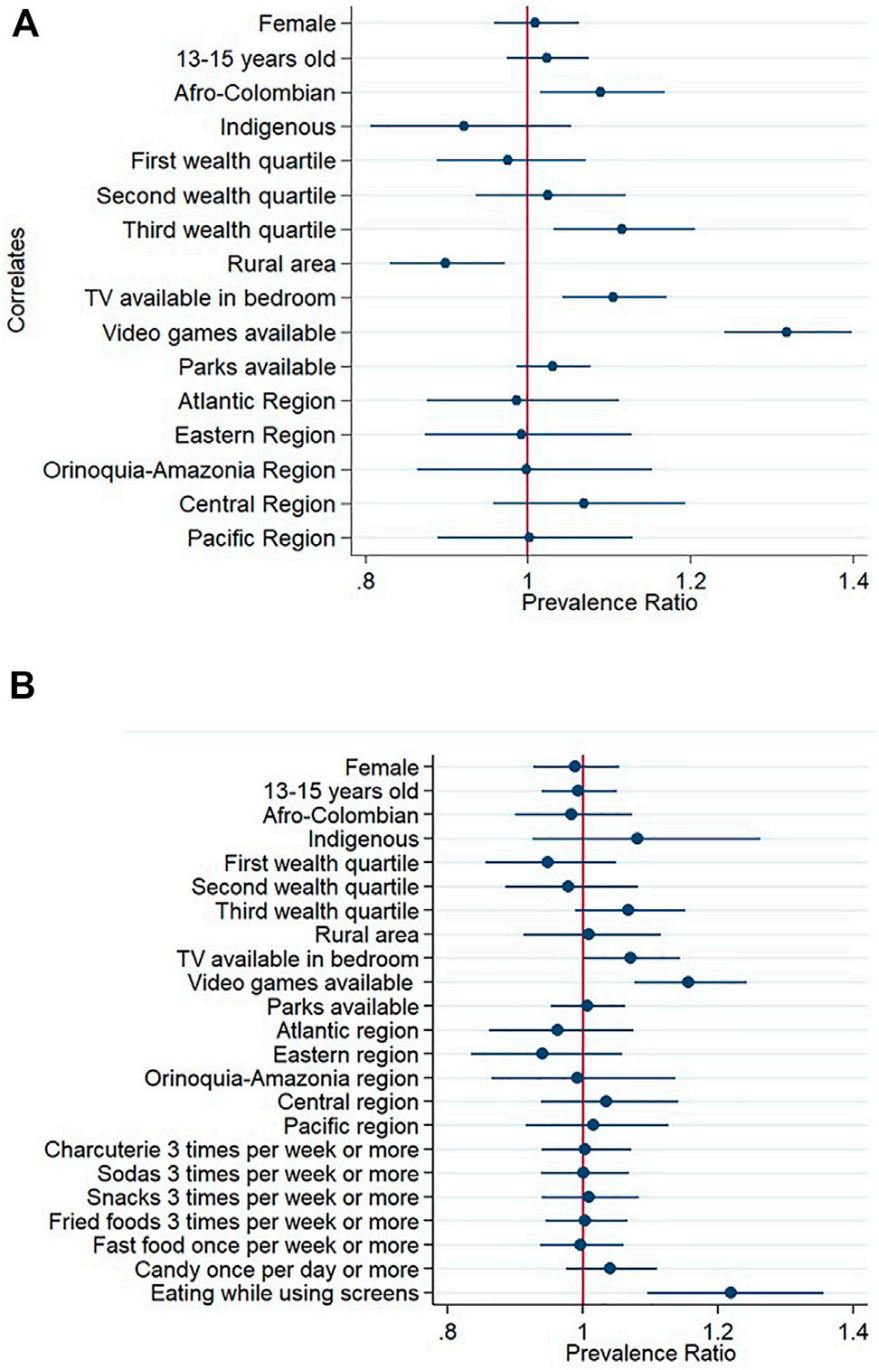
Prevalence ratios and 95% confidence intervals of associated factors of spending excessive recreational screen-time among Colombian adolescents. Excessive screen-time was defined as spending more than 2 h of screen-time per day. **(A)** Mutivariable model 1 **(B)** Mutivariable model 2 including food consumption variables. Reference categories were male sex, 16–17 years of age, no ethnic identity reported, fourth wealth quartile, urban area, no TV availability, no video games availability, no parks availability, Capital District region, no consumption of the food items included and not eating while using screens. National Survey of Nutrition, Colombia, 2015.

According to multivariable model 1, Afro-Colombian adolescents, those in the third wealth index quartile and those who had TV available in their rooms or video-games available were more likely to have excessive RST than their counterparts. On the contrary, those living in rural areas were less likely to have excessive RST than their counterparts (Figure 3A; Supplementary Table 3). Program participation variables were not associated in the bivariate models and therefore were not included in model 2. According to multivariable model 2, adolescents who usually eat in front of screens were more likely to have excessive RST (Figure 3B; Supplementary Table 3).

## Discussion

Our results indicate that a large proportion of Colombian children and adolescents have excessive RST and the prevalence increases with age. Approximately five out of every 10 preschoolers, six out of every 10 school-aged children, and seven out of every 10 adolescents had excessive RST. Associated factors differed across age groups, but consistent positive associations were observed with the availability of TV in the child’s bedroom, the availability of video games, and eating while using screens. Also, a negative association with rural area was observed for all age groups. Factors at the intrapersonal, household and environmental levels are discussed below.

At the intrapersonal level RST associated factors included age, ethnicity, consumption of energy dense foods and food intake while using screens. Younger preschoolers (3–4 years old) and older school-aged children (10–12 years old) were more likely to have excessive RST. Our findings for school-aged children are consistent with other studies that have reported a positive association between age and screen time [29, 30]. However, among preschoolers, our results, which contribute to the limited studies on this population, showed an opposite association, which can be related to the fact that the sedentary behavior guidelines used as cut-offs in this analysis are stricter for younger children [31]. It is important that public health messages consider this difference in the sedentary behavior guidelines according to age. The observed associations with ethnicity suggest that children belonging to Indigenous communities use screens less and this situation should be preserved. On the contrary, Afro-Colombian adolescents showed an increased likelihood of having excessive RST, which indicates that initiatives or programs to decrease sedentary behaviors should have a special focus on this population and further research is needed to better understand the drivers of this association. Our findings for food consumption are aligned with other studies, showing that children and adolescents with excessive RST have a greater consumption of energy dense foods and drinks [32] and tend to eat their meals while using screens [32, 33]. These associations are concerning considering that these obesogenic behaviors are present at a high proportion from early ages. Specific public health messaging for parents and children should be designed not only to reduce RST but also to avoid practices like eating while watching TV or other screens. The dietary guidelines of Brazil, for example, encourage sitting with the family to eat meals in an appropriate environment paying full attention to the act of eating and without engaging in another activity [34].

At the household level, living in a rural area and electronic devices availability emerged as consistent associated factors across all age groups. The negative association with rural area was a common correlate [17, 35] that highlights the importance of focusing interventions on populations living in the continuously growing urban areas. The association of RST and sitting time with devices availability has been previously reported, mainly for school-aged children and adolescents [35–37]. In our study we observed that this is a relevant correlate from early age, which is a reason for concern considering that TV availability in the bedroom contributes to an obesogenic environment [38]. Future actions should take into account that having a TV in the bedroom and video-game availability are modifiable factors, and removing them from children’s bedrooms can contribute to limiting screen time, as demonstrated by Atkin et al. [39] in a longitudinal study. Our results also indicate that the wealthiest children and adolescents are more likely to have excessive RST than their poorer counterparts. This association was also reported in a systematic review and meta-analysis that found a positive relationship between socioeconomic level and excessive RST in adolescents form low-middle income countries, and opposite to the association observed in high income countries [40]. These different patterns in the association between RST and socioeconomic level resemble the patterns proposed by the obesity transition stages, where earlier stages are characterized by higher prevalence, in this case of obesity, among the wealthiest people and later stages show higher prevalence among those in the lowest socioeconomic status [41]. These results can suggest a lifestyle transition that require urgent action to reduce the high prevalence of RST among the wealthiest children and to prevent the increase of these behaviors among the most vulnerable ones.

At the environmental level, we did not find a statistically significant association of RST with the availability of physical activity programs. Previous evidence on Ciclovía participation, showed that children who attended this program had lower total sedentary time on sundays, however RST was not specifically assessed in this study [42]. The lack of associations observed in our results can be understood by the fact that the programs included in the survey are focused on physical activity promotion, and may indicate the need to have specific interventions or strategies aimed at reducing RST together with the already existing physical activity programs to have an impact on the children and adolescent’s movement behaviors in Colombia. In this regard, it is important to highlight that there is a lack of policies and interventions to reduce screen time among Colombian children and adolescents, as previously reported by a group of experts in the Colombian Report Card of Physical Activity [43]. We also observed a counter-intuitive positive association of excessive RST with parks availability. This finding could be related to safety conditions of the existing parks, as suggested by the non-statistically significant positive association between excessive RST and lack of safety perception observed in the bivariate analysis for preschoolers and school-aged children. In terms of geographic region, we also observed that preschoolers from the Atlantic and Pacific regions, and adolescents from the Atlantic region were less likely to have excessive RST compared to their counterparts from Bogotá (the capital district). This could be probably influenced by differences in internet accessibility and cultural norms on the use of leisure time that should be further studied.

Our estimates seem higher than the prevalence of excessive RST reported in previous versions of the ENSIN survey [17]. However, it is important to mention that our results may not be comparable, since different questionnaires have been used and the most recent version of ENSIN includes a wider range of screen-devices. In the global context, our results are similar to the engagement in RST reported by very high-income countries like Canada, Denmark, England and Qatar [6]. Having a prevalence comparable to those observed in rich countries may be indicative of the lifestyle transition that low- and middle-income countries are experiencing and reinforces the urgent need to proactively implement actions to decrease risk behaviors such as excessive RST [44]. In the current context of the COVID-19 pandemic that has drastically changed the routine and movement behaviors of children and adolescents it would be expected that the observed estimates have largely increased, as observed in other countries [45]. This adds to the urgency for the design and implementation of strategies to reduce RST. These actions should align with the call made by the WHO-UNICEF- Lancet Commission to place children as the center of the Sustainable Development Goals. Specifically, greater regulation of advertising and commercial governance to protect children from the exposure to marketing of unhealthy products and harmful contents of videogames should be priorities for decision-makers [46].

Our results should be interpreted considering strengths and limitations of the study. The main strength is that our estimates inform the situation of RST for a nationally representative sample of 3- to 17-year-old individuals from Colombia, the broadest age range evaluated at the national level, to the best of our knowledge. Also, the survey data used for this analysis provides a wide range of contextual covariates of relevance to understand the RST situation in Colombia. Limitations include the cross-sectional design of the survey, which does not allow to make causal inferences from our findings, and the use of self- and proxy-report of screen time engagement. Despite self-report being the most feasible method of assessment of RST for population surveys, it is important to recognize that this measure of screen time can be influenced by social desirability bias and recall bias and may lead to a certain degree of misclassification. Also, the questionnaire used to ascertain engagement in RST did not allow for a continuous variable of the total time engaged in RST. In addition, the questionnaire used for preschoolers showed a low internal consistency for the sedentary behaviors section [20], which suggests low agreement between the items to assess the sedentary behaviors construct. However, for the analyses conducted in this paper, not all of the items were taken into account given that the variable of interest was RST and not sedentary behaviors as a broad construct. Finally, RST at the school setting may be underestimated, since the questionnaire used for preschoolers only inquired about activities conducted out of the school setting. For school-aged children and adolescents, the context of the screen time-related activities was not assessed, but the questions used inquired exclusively about non schoolwork-related activities.

In conclusion, the majority of Colombian children and adolescents have excessive RST and several factors were identified that can be considered in the design of strategies to decrease these behaviors. The non-modifiable factors identified in this study can guide interventions aimed at decreasing RST among children and youth, for target populations such as younger preschoolers, older school-aged children, children and adolescents from urban areas and the wealthiest children and adolescents. Significant modifiable factors, such as availability of electronic devices, provide guidance for promising strategies that can be considered as part of a comprehensive initiative to reduce RST, such a public health campaigns to reduce the availability of TVs and electronic devices in children’s bedrooms and not eating in front of the TV.
