# Supplementary material for: Prevalence and Associated Factors of Excessive Recreational Screen Time Among Colombian Children and Adolescents
Source: Int J Public Health. 2022 Feb 23;67:1604217. doi: 10.3389/ijph.2022.1604217 (PMC8904350; doi:10.3389/ijph.2022.1604217)
Supplement: Supplementary file 2 [file Table2.docx]

Supplementary table 2 Associated factors of excessive recreational screen time among Colombian school-aged children. National Survey of Nutrition, Colombia 2015.

| **Sociodemographic and other potentially relevant variables for screen-time** | **Model 1 ^a^** | | |  | **Model 2 ^b^** | | |  |
| --- | --- | --- | --- | --- | --- | --- | --- | --- |
|  | **PR** | **CI** | **p-value** |  | **PR** | **CI** | **p-value** |  |
| **Sex** |  |  |  |  |  |  |  |  |
| Female | 0.99 | (0.9-1.1) | 0.75 |  | 1.01 | (0.9-1.1) | 0.895 |  |
| Male | 1.00 |  |  |  | 1.00 |  |  |  |
| **Age** |  |  |  |  |  |  |  |  |
| 6-9 | 0.91 | (0.9-1.0) | 0.006 |  | 0.93 | (0.9-1.0) | 0.063 |  |
| 10-12 | 1.00 |  |  |  | 1.00 |  |  |  |
| **Ethnicity** |  |  |  |  |  |  |  |  |
| Afro-colombian | 0.97 | (0.9-1.1) | 0.693 |  | 0.94 | (0.8-1.1) | 0.396 |  |
| Indigenous | 0.62 | (0.4-0.9) | 0.007 |  | 0.77 | (0.5-1.2) | 0.273 |  |
| No ethnic identity reported | 1.00 |  |  |  | 1.00 |  |  |  |
| **Overweight** |  |  |  |  |  |  |  |  |
| Yes | 1.06 | (1.0-1.1) | 0.081 |  | 1.03 | (0.9-1.1) | 0.511 |  |
| No | 1.00 |  | - |  | 1.00 |  |  |  |
| **Obesity** |  |  |  |  |  |  |  |  |
| Yes | 1.04 | (0.9-1.1) | 0.948 |  | 0.92 | (0.8-1.1) | 0.260 |  |
| No | 1.00 |  |  |  | 1.00 |  |  |  |
| **Participation in organized activities and programs** |  |  |  |  |  |  |  |  |
| Ciclovias ^c^ |  |  |  |  |  |  |  |  |
| Yes | - | - | - |  | 1.01 | (0.8-1.2) | 0.936 |  |
| No | - |  |  |  | 1.00 |  |  |  |
| Sport Clubs |  |  |  |  |  |  |  |  |
| Yes | - | - | - |  | 1.00 | (0.9-1.1) | 0.985 |  |
| No | - |  |  |  | 1.00 |  |  |  |
| Organized groups (dance, martial arts, etc) |  |  |  |  |  |  |  |  |
| Yes | - | - | - |  | 1.10 | (1.0-1.2) | 0.051 |  |
| No | - |  |  |  | 1.00 |  |  |  |
| **Physically active ^d^** |  |  |  |  |  |  |  |  |
| Yes | 0.95 | (0.9-1.0) | 0.189 |  | 1.02 | (0.9-1.1) | 0.744 |  |
| No | 1.00 |  |  |  | 1.00 |  |  |  |
| **Food intake** |  |  |  |  |  |  |  |  |
| ***Charcuterie 3 times per week or more*** | |  |  |  |  |  |  |  |
| Yes | - | - | - |  | 1.07 | (1.0-1.2) | 0.064 |  |
| No | - |  |  |  | 1.00 |  |  |  |
| ***Sodas 3 times per week or more*** | |  |  |  |  |  |  |  |
| Yes | - | - | - |  | 1.05 | (1.0-1.1) | 0.253 |  |
| No | - |  |  |  | 1.00 |  |  |  |
| ***Snacks 3 times per week or more*** | |  |  |  |  |  |  |  |
| Yes | - | - | - |  | 0.98 | (0.9-1.1) | 0.675 |  |
| No | - |  |  |  | 1.00 |  |  |  |
| ***Fried foods 3 times per week or more*** | |  |  |  |  |  |  |  |
| Yes | - | - | - |  | 1.08 | (1.0-1.2) | 0.083 |  |
| No | - |  |  |  | 1.00 |  |  |  |
| ***Candy once per day or more*** |  |  |  |  |  |  |  |  |
| Yes | - | - | - |  | 0.99 | (0.9-1.1) | 0.761 |  |
| No | - |  |  |  | 1.00 |  |  |  |
| ***Usually eats while using screens*** | |  |  |  |  |  |  |  |
| Yes | - | - | - |  | 1.38 | (1.2-1.6) | <0.001 |  |
| No | - |  |  |  | 1.00 |  |  |  |
| **Wealth Quartiles** |  |  |  |  |  |  |  |  |
| First (poorest) | 0.85 | (0.7-1.0) | 0.032 |  | 0.81 | (0.7-1.0) | 0.016 |  |
| Second | 1.00 | (0.9-1.1) | 0.990 |  | 0.95 | (0.8-1.1) | 0.452 |  |
| Third | 1.00 | (0.9-1.1) | 0.949 |  | 0.98 | (0.9-1.1) | 0.739 |  |
| Fourth (wealthiest) | 1.00 |  |  |  | 1.00 |  |  |  |
| **Area** |  |  |  |  |  |  |  |  |
| Urban | 1.00 |  |  |  | 1.00 |  |  |  |
| Rural | 0.88 | (0.8-1.0) | 0.035 |  | 1.01 | (0.9-1.2) | 0.923 |  |
| **TV available at the child's bedroom** | |  |  |  |  |  |  |  |
| Yes | 1.13 | (1.1-1.2) | 0.001 |  | 1.08 | (1.0-1.2) | 0.098 |  |
| No | 1.00 |  |  |  | 1.00 |  |  |  |
| **Videogames available** |  |  |  |  |  |  |  |  |
| Yes | 1.26 | (1.2-1.4) | <0.001 |  | 1.21 | (1.1-1.3) | <0.001 |  |
| No | 1.00 |  |  |  | 1.00 |  |  |  |
| **Parks availability in the neighborhood** | |  |  |  |  |  |  |  |
| Yes | 1.11 | (1.0-1.2) | 0.015 |  | 1.05 | (1.0-1.2) | 0.259 |  |
| No | 1.00 |  |  |  | 1.00 |  |  |  |
| **Geographic region** |  |  |  |  |  |  |  |  |
| Atlantic | 0.92 | (0.8-1.1) | 0.300 |  | 0.88 | (0.7-1.1) | 0.159 |  |
| Eastern | 1.07 | (0.9-1.2) | 0.257 |  | 0.97 | (0.9-1.1) | 0.630 |  |
| Orinoquia-Amazonia | 0.97 | (0.8-1.1) | 0.693 |  | 0.97 | (0.8-1.1) | 0.650 |  |
| Capital District | 1.00 |  |  |  | 1.00 |  |  |  |
| Central | 1.03 | (0.9-1.2) | 0.651 |  | 0.94 | (0.8-1.1) | 0.316 |  |
| Pacific | 1.07 | (0.9-1.3) | 0.371 |  | 0.98 | (0.8-1.2) | 0.803 |  |
| Abbreviations: PR, prevalence ratio; CI, 95% confidence interval | | | | | | | |  |
| a Model 1 for school-aged children included sex, age, ethnicity, overweight, physical activity, wealth index, area of residence, TV availability in the child's bedroom, video games available at home, parks availability in the neighbourhood and geographic region. | | | | | | | | |
| b Model 2 for school-aged children included sex, age, ethnicity, overweight, participation in Ciclovías, sports clubs and organized groups, physical activity, food intake variables, wealth index, area of residence, TV availability in the child's bedroom, video games available at home, parks availability in the neighbourhood and geographic region. | | | | | | | | |
| c Ciclovias are defined as a program that closes the streets to motorized vehicles, usually Sundays and holidays, for recreational and exercise purposes. | | | | | | | | |
| d Physically active is defined as meeting the WHO physical activity guidelines | | | | | | |  |  |
