# Supplementary material for: Prevalence and Associated Factors of Excessive Recreational Screen Time Among Colombian Children and Adolescents
Source: Int J Public Health. 2022 Feb 23;67:1604217. doi: 10.3389/ijph.2022.1604217 (PMC8904350; doi:10.3389/ijph.2022.1604217)
Supplement: Supplementary file 3 [file Table3.docx]

Supplementary table 3 Associated factors of recreational screen time among Colombian adolescents. National Survey of Nutrition, Colombia 2015

| **Sociodemographic and other potentially relevant variables for screen-time** | **Model 1 ^a^** | | |  | **Model 2 ^b^** | | |
| --- | --- | --- | --- | --- | --- | --- | --- |
|  | **PR** | **CI** | **p-value** |  | **PR** | **CI** | **p-value** |
| **Sex** |  |  |  |  |  |  |  |
| Female | 1.01 | (1.0-1.1) | 0.721 |  | 0.98 | (0.9-1.1) | 0.714 |
| Male | 1.00 |  |  |  | 1.00 |  |  |
| **Age** |  |  |  |  |  |  |  |
| 13-15 | 1.02 | (1.0-1.1) | 0.358 |  | 0.99 | (0.9-1.0) | 0.801 |
| 16-17 | 0.00 |  |  |  | 1.00 |  |  |
| **Ethnicity** |  |  |  |  |  |  |  |
| Afro-colombian | 1.09 | (1.0-1.2) | 0.018 |  | 0.98 | (0.9-1.1) | 0.692 |
| Indigenous | 0.92 | (0.8-1.1) | 0.226 |  | 1.08 | (0.9-1.3) | 0.318 |
| No ethnic identity reported | 1.00 |  |  |  | 1.00 |  |  |
| **Obesity** |  |  |  |  |  |  |  |
| Yes | 1.05 | (0.9-1.1) | 0.278 |  | 1.01 | (0.9-1.2) | 0.861 |
| No | 1.00 |  |  |  | 1.00 |  |  |
| **Food intake** |  |  |  |  |  |  |  |
| ***Charcuterie 3 times per week or more*** |  |  |  |  |  |  |  |
| Yes | - | - | - |  | 1.00 | (0.9-1.1) | 0.919 |
| No | - |  |  |  | 1.00 |  |  |
| ***Sodas 3 times per week or more*** |  |  |  |  |  |  |  |
| Yes | - | - | - |  | 1.00 | (0.9-1.1) | 0.974 |
| No | - |  |  |  | 1.00 |  |  |
| ***Snacks 3 times per week or more*** |  |  |  |  |  |  |  |
| Yes | - | - | - |  | 1.01 | (0.9-1.1) | 0.815 |
| No | - |  |  |  | 1.00 |  |  |
| ***Fried foods 3 times per week or more*** |  |  |  |  |  |  |  |
| Yes | - | - | - |  | 1.00 | (0.9-1.1) | 0.911 |
| No | - |  |  |  | 1.00 |  |  |
| ***Fast food once per week or more*** |  |  |  |  |  |  |  |
| Yes | - | - | - |  | 1.00 | (0.9-1.1) | 0.922 |
| No | - |  |  |  | 1.00 |  |  |
| ***Candy once per day or more*** |  |  |  |  |  |  |  |
| Yes | - | - | - |  | 1.04 | (1.0-1.1) | 0.227 |
| No | - |  |  |  | 1.00 |  |  |
| ***Usually eats while using screens*** |  |  |  |  |  |  |  |
| Yes |  |  |  |  | 1.22 | (1.1-1.4) | <0.001 |
| No |  |  |  |  | 1.00 |  |  |
| **Wealth Quartiles** |  |  |  |  |  |  |  |
| First (poorest) | 0.98 | (0.9-1.1) | 0.599 |  | 0.95 | (0.9-1.0) | 0.294 |
| Second | 1.02 | (0.9-1.1) | 0.597 |  | 0.98 | (0.9-1.1) | 0.669 |
| Third | 1.12 | (1.0-1.2) | 0.007 |  | 1.07 | (1.0-1.2) | 0.094 |
| Fourth (wealthiest) | 1.00 |  |  |  | 1.00 |  |  |
| **Area** |  |  |  |  |  |  |  |
| Urban | 1.00 |  |  |  | 1.00 |  | - |
| Rural | 0.90 | (0.8-1.0) | 0.008 |  | 1.01 | (0.9-1.1) | 0.860 |
| **TV available at the child's bedroom** |  |  |  |  |  |  |  |
| Yes | 1.11 | (1.0-1.2) | 0.001 |  | 1.07 | (1.0-1.1) | 0.046 |
| No | 1.00 |  |  |  | 1.00 |  |  |
| **Videogames available** |  |  |  |  |  |  |  |
| Yes | 1.32 | (1.2-1.4) | <0.001 |  | 1.16 | (1.1-1.2) | <0.001 |
| No | 1.00 |  |  |  | 1.00 |  |  |
| **Parks availability in the neighborhood** |  |  |  |  |  |  |  |
| Yes | 1.03 | (1.0-1.1) | 0.168 |  | 1.01 | (1.0-1.1) | 0.827 |
| No | 1.00 |  |  |  | 1.00 |  |  |
| **Geographic region** |  |  |  |  |  |  |  |
| Atlantic | 0.99 | (0.9-1.1) | 0.825 |  | 0.96 | (0.9-1.1) | 0.493 |
| Eastern | 0.99 | (0.9-1.1) | 0.905 |  | 0.94 | (0.8-1.1) | 0.299 |
| Orinoquia-Amazonia | 1.00 | (0.9-1.2) | 0.980 |  | 0.99 | (0.9-1.1) | 0.903 |
| Capital District | 1.00 |  |  |  | 1.00 |  |  |
| Central | 1.07 | (1.0-1.2) | 0.227 |  | 1.04 | (0.9-1.1) | 0.483 |
| Pacific | 1.00 | (0.9-1.1) | 0.976 |  | 1.02 | (0.9-1.1) | 0.760 |
| Abbreviations: PR, prevalence ratio; CI, 95% confidence intervals | | | | | | | |
| a Model 1 for adolescents included sex, age, ethnicity, wealth index, area of residence, TV availability in the child's bedroom, video games available at home, parks availability in the neighbourhood and geographic region. | | | | | | | |
| b Model 2 for adolescents included sex, age, ethnicity, food intake variables, wealth index, area of residence, TV availability in the child's bedroom, video games available at home, parks availability in the neighbourhood and geographic region. | | | | | | | |
